# Acute Effects of Loaded and Unloaded Whole-Body Vibration on Vertical Jump Performance in Karate Athletes

**DOI:** 10.5114/jhk/172637

**Published:** 2023-11-28

**Authors:** Haris Pojskic, Željko Zombra, Jad Adrian Washif, Jeffrey Pagaduan

**Affiliations:** 1 Department of Sports Science, Faculty of Social Sciences, Linnaeus University, Kalmar, Sweden.; 2 Institute for Kinesiology, Lukavac, Bosnia and Herzegovina.; 3 Faculty of Physical Education and Sports, University of Tuzla, Bosnia and Herzegovina.; 4 Sports Performance Division, Institut Sukan Negara Malaysia (National Sports Institute of Malaysia), Kuala Lumpur, Malaysia.; 5 Faculty of Physical Culture, Palacký University Olomouc, Olomouc, Czech Republic.

**Keywords:** martial arts, countermovement jump, explosive power, isometric contraction, postactivation potentiation enhancement

## Abstract

We investigated the acute effects of different whole-body vibration (WBV) interventions on the jump height of highly trained karate practitioners. Fifteen male karate club athletes (age: 20.0 ± 3.8 years; stature: 177.3 ± 4.7 cm; body mass: 76.9 ± 11.2 kg; % body fat: 9.2 ± 4.3) performed six randomized interventions: [a] static half-squat (SHS); [b] SHS with external loads at 30% of the body weight (SHS + 30%BW); [c] WBV at frequency (f) 25 Hz, and 2 mm amplitude (A) (WBV 25/2); [d] WBV 25/2 with external loads of 30% of the body weight (WBV 25/2 + 30% BW); [e] WBV at f = 50 Hz, and A = 4 mm (WBV 50/4), and [f] WBV 50/4 with external loads of 30% of the body weight (WBV 50/4 + 30% BW). Each intervention was performed for 5 sets at 60 s/set, with a rest interval of 30 s between sets. Countermovement jump (CMJ) data were collected at 2, 4, 6, 8 and 10 min after each preconditioning intervention. Two-way repeated-measures ANOVA revealed a non-significant main effect of intervention [F(5, 10) = 1.44, η2 = 0.42, p = 0.29)] and a significant main effect of the rest interval [F(4, 11) = 3.51, η2 = 0.56, p = 0.04)] on CMJ height. A rest interval of 4 min resulted in significantly higher CMJ values than a rest interval of 2 min (p = 0.031). In conclusion, utilizing a 4-min rest interval irrespective of the intervention schemes may have potential for enhancing jumping performance among highly trained karate athletes.

## Introduction

Explosive power is an important component in karate performance, especially in “kumite”, which is a free form of fighting (Roschel et al., 2009). Efficient karate punching, kicking and rapid movements are manifestations of well-developed muscular power (Martínez de Quel et al., 2020), facilitated by a good warm-up. A typical karate warm-up consists of an aerobic activity followed by dynamic and passive stretching exercises. Recently, there has been increasing interest in designing warm-up strategies to elicit power. Among these is whole-body vibration (WBV).

Several mechanisms are believed to enhance performance from WBV (Bullock et al., 2008; Dallas et al., 2023; Pojskic et al., 2015). WBV can produce a sine wave motion from the vibration’s acceleration (intensity), amplitude, and frequency, which prompts muscles and tendons to cyclically lengthen and shorten, resulting in a rapid and energy-efficient warm-up effect (Washif, 2019). Thus, WBV is thought to produce compensatory muscular contractions from mechanical vibrations via stimulation of primary endings of muscle spindles and activation of alpha-motor neurons, resulting in higher power output (Cardinale and Bosco, 2003; Di Iorio et al., 2012). Furthermore, WBV has also been demonstrated to improve intra- and intermuscular coordination, as well as motor unit recruitment and synchronization (Cardinale and Lim, 2003). Additionally, WBV is also linked to post-activation potentiation enhancement (PAPE), which is a combination of various physiological conduits contributing to increased muscle output from preload stimulation (Berriel et al., 2022; Blazevich and Babault, 2019; Garbisu-Hualde et al., 2023; Krzysztofik and Wilk, 2020). With these characteristics, WBV appears to be a promising approach to optimize the warm-up.

Different mechanical stimuli exist in WBV. Researchers suggested WBV set-up with amplitudes between 4 mm and 10 mm, exposure duration from 30 s to 4 min, a work-to-rest ratio of 1:1 to 1:3, and rest intervals from 0 to 15 min to elicit positive benefits (Dabbs et al., 2012; Dallas et al., 2015). Furthermore, when applying 50 Hz of frequency, a 4 mm to 6 mm amplitude is recommended (Dabbs et al., 2012; Dallas et al., 2015). However, for highly trained individuals, additional external loads may be necessary during WBV to further optimize force production (Pojskic et al., 2015; Rønnestad, 2009). For example, previous studies indicated that an additional external load of 30% of the body weight under WBV posted superior gains in the countermovement jump, speed and agility compared to unloaded WBV, loaded non-WBV and unloaded non-WBV interventions in well-trained college soccer players (Pojskic et al., 2015).

Rest intervals also affect performance with WBV. Sale (2004) suggests that rest intervals should reach a balance between allowing the individual to fully recover from fatigue, and ensuring that the acute potentiating effects on neuromuscular function, induced by the loading stimulus, are not yet diminished. There exists a lack of consensus about the most appropriate time of rest following a single session of WBV exercises, as evidenced by conflicting findings reported in different studies (Adams et al., 2009; Bedient et al., 2009; Cochrane and Booker, 2014; Dabbs et al., 2011; Dallas et al., 2015; Kostrzewa et al., 2020). Those studies have documented the positive impact of WBV on the vertical jump height within time intervals ranging from 0 to 15 min.

Athletes and coaches constantly attempt to find ways to improve performance from a warm-up (Washif, 2019). At present, a limited number of studies have attempted to assess vibration frequency, amplitude, and the external load in WBV (Naclerio et al., 2014). To the best of the researchers' knowledge, no investigations on warm-up settings employing loaded and unloaded WBV in karate practitioners have been conducted. WBV as a warm-up in karate might be an effective strategy to increase leg power to improve vertical jump height. Therefore, the purpose of this study was to examine the effects of loaded and unloaded WBV schemes with varying rest intervals (2, 4, 6, 8 and 10 min) on countermovement jump (CMJ) performance. We hypothesized that WBV with an additional load would generate the highest gains in CMJ performance compared to the results of the other preconditioning protocols.

## Methods

### Participants

Fifteen highly trained male karate practitioners (age: 20.0 ± 3.8 years; stature: 177.3 ± 4.7 cm; body mass: 76.9 ± 11.2 kg; % body fat: 9.23 ± 4.3) from several karate clubs volunteered to participate in the study. All had no history of musculoskeletal injuries within 6 months prior to data collection. They competed at the international (n = 7) and national (n = 8) levels and had 10.2 ± 3.2 years of training and 8.2 ± 3.1 years of competitive experience. According to their coaches, they trained 7.5 h a week (5 sessions of 1.5 h each) on tatami to improve technical and tactical skills, and 3 h a week (2 sessions of 1.5 h each) in a weight room to develop strength, power, and endurance. A light meal was allowed to be consumed >3 h before the beginning of testing sessions. To maintain hydration status and avoid excessive dehydration, athletes were allowed to drink the appropriate amount of water during testing sessions (Chryssanthopoulos et al., 2023). They were encouraged to avoid strenuous activity, tobacco, alcohol consumption, and caffeine intake 48 h prior to the testing sessions. Non-complying athletes, including those who reported having sleep deprivation, were excluded. Prior to data collection, all athletes were informed about the potential benefits and risks associated with the study. They all provided written informed consent. This study was approved by the Institutional Review Board of the Faculty of Physical Education and Sports, University of Tuzla (02/11-2370/13-5.5.) with procedures conforming to the principles of the Declaration of Helsinki on human experimentation.

### Design and Procedures

This cross-sectional study investigated the effects of acute loaded and unloaded WBV on CMJ performance. Participants were exposed to various loaded and unloaded warm-up schemes in randomized order. Post-intervention CMJs at different time points were gathered to identify an appropriate rest interval between stimulus and performance.. All participants in the current study visited the Exercise Science Laboratory for 7 sessions between 8 a.m. and 10 a.m., separated by 48 h. The first session was assigned for the measurement of stature, body mass, and body fat content. A handheld stadiometer (Astra scale 27310, Gima, Italy) was used to assess stature at nearest 1 cm. A bioelectric body composition analyzer (Tanita TBF-300 increments 0.1%; Tanita, Tokyo, Japan) was utilized to evaluate body mass and body fat content. Testing equipment and experimental procedure familiarization were also conducted during that session. Then, participants underwent randomization to determine the order of schemes in the next 6 sessions. All randomized interventions were preceded by a 5-min warm-up on a cycle ergometer at 125 W and 70–75 rpm (Life Fitness, USA).

Warm-up schemes involved the following: (i) a static half squat (SHS); (ii) a SHS with an additional external load of 30% of the body weight (SHS + 30%BW); (iii) whole-body vibration at *f* = 25 Hz, A = 2 mm (WBV 25/2); (iv) WBV 25/2 with an additional external load of 30% of the body weight (WBV 25/2 + 30% BW); (v) whole-body vibration at *f* = 50 Hz, A = 4 mm (WBV 50/4); and (vi) WBV 50/4 with an additional external load of 30% of the body weight (WBV 50/4 + 30% BW). All interventions were carried out 5 times for 60 s for a total of 5 min, with a rest interval of 30 s between sets. Participants stepped off the platform (POWRX^®^ Vibration Plate Pro Evolution 2.7, Germany) and stood for 30 s during rest in WBV schemes. The squat pattern followed a 100-degree knee flexion measurement with feet slightly wider than shoulder width apart. For loaded schemes, an Olympic bar and an appropriate set of weights were applied to achieve extra 30% of the BW.

**Figure 1:**
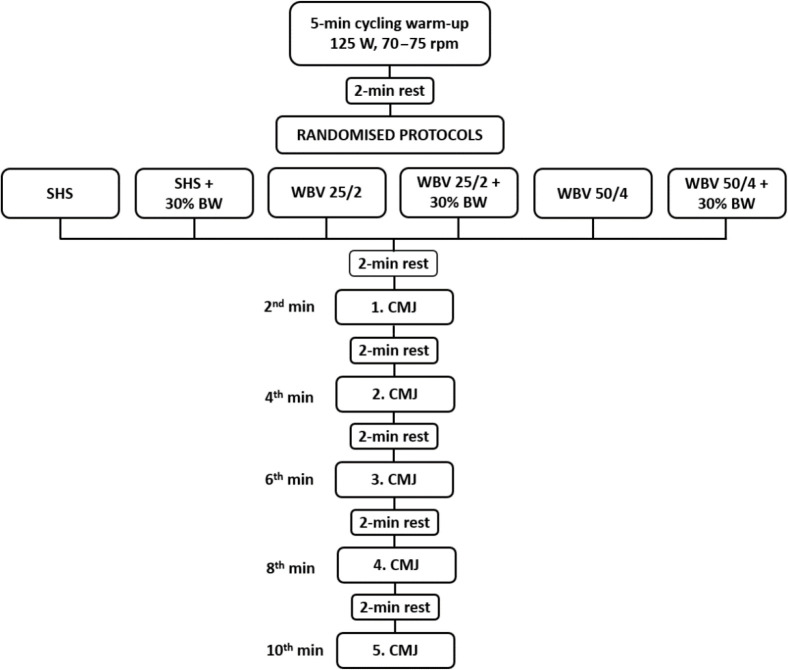
Experimental protocol. CMJ: Countermovement jump, rpm: revolutions per minute, WBV: whole-body vibration, SHS: standing in a static half squat position, SHS + 30% BW: SHS with an additional load of 30% of the body weight, WBV 25/2: WBV at f = 25 Hz, A = 2 mm, WBV 25/2 + 30% BW: WBV 25/2 with an additional load of 30% of the body weight, WBV 50/4: WBV at f = 50 Hz, A = 4 mm, WBV 50/4 + 30% BW: WBV 50/4 with an additional load of 30% of the body weight.

CMJ data were collected at 2, 4, 6, 8 and 10 min after each conditioning stimulus. At each time point, athletes performed two CMJs, separated by a 5-s rest interval. Participants were seated during the recovery periods. During the CMJ, participants started in an upright position with hands akimbo, to avoid arm swings throughout the vertical jump. A rapid downward movement (approximately semi-squat level) was then executed, and followed by an upward maximal jump. Athletes were instructed to land at the same starting spot, while bending the knees upon ground contact to reduce mechanical stress (Pojskic et al., 2022; Washif and Kok, 2022). The CMJ was conducted using commercial equipment (Optojump System, Microgate, Bolzano, Italy), with the best trial used for further analysis. The experimental protocol is displayed in Figure 1.

**Figure 2:**
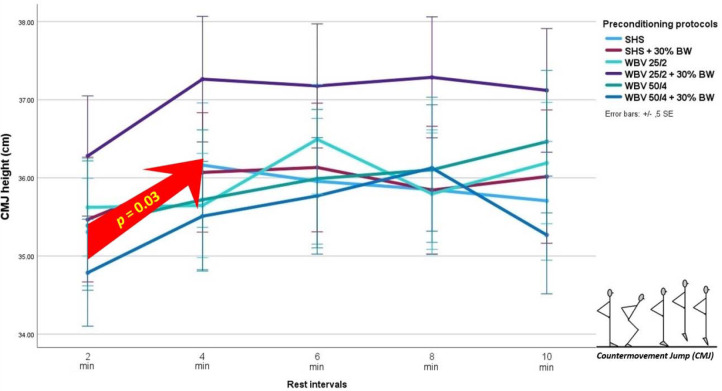
Multiple comparison of different loaded and unloaded whole-body vibration (WBV) and low intensity intermittent isometric interventions at various recovery intervals on countermovement jump (CMJ) height. SHS: standing in a static half squat position; SHS + 30% BW: SHS with an additional load of 30% of the body weight, WBV 25/2: WBV at f = 25 Hz, A = 2 mm, WBV 25/2 + 30% BW: WBV 25/2 with an additional load of 30% of the body weight, WBV 50/4: WBV at f = 50 Hz, A = 4 mm, WBV 50/4 + 30% BW: WBV 50/4 with an additional load of 30% of the body weight. The red arrow shows significant improvement in CMJ height after 4-min recovery compared to 2-min recovery (*p* = 0.03).

### Statistical Analysis

All analyses were performed using commercial software SPSS^®^ 24.0 for Windows (IBM Statistics, Chicago, IL) with alpha threshold set at 0.05. Data are presented with the means as well as standard deviations (Table 1). The Shapiro-Wilk test was used to verify data normality. A two-way repeated-measures ANOVA with six interventions x five recovery intervals (6 x 5) was carried out to determine any significant differences in vertical jump performance across the interventions and recovery intervals. The Mauchly’s test was utilized to examine the sphericity of the data (Field, 2009). Eta squared (η^2^) was used to estimate the effect size. The Bonferroni correction was applied to assess the significance of the post hoc pairwise comparisons. To calculate the smallest worthwhile change (SWC) considering CMJ height, average between-subject standard deviation (SD) of the all warm-up schemes was multiplied by 0.2, which represents the typical small effect (Pyne et al., 2005). Before the commencement of the study, sample size was determined using G-Power software (version 3.1.9.2; Heinrich Heine University Dusseldorf, Dusseldorf, Germany). For repeated measures ANOVA (*p* value of 0.05, effect size of 0.30, five measurements and power of 0.80), the program estimated 15 subjects (df = 13) as an appropriate sample size.

**Table 1:** Descriptive variables (means ± SD) of countermovement jump (CMJ) height (cm) after different conditioning interventions and recovery intervals.

Recovery intervals	PRECONDITIONING INTERVENTIONS						
	SHS	SHS + 30% BW	WBV 25/2	WBV 25/2 + 30% BW	WBV 50/4	WBV 50/4 + 30% BW	Average
2 min	35.3 ± 5.3	35.5 ± 6.2	35.6 ± 4.8	36.3 ± 6.0	35.4 ± 6.4	34.8 ± 5.3	35.5 ± 5.4
4 min	36.2 ± 6.2	36.1 ± 5.9	35.6 ± 5.2	37.3 ± 6.2	35.7 ± 6.9	35.5 ± 5.4	36.1 ± 5.8^*^
6 min	35.9 ± 6.2	36.1 ± 6.4	36.5 ± 5.4	37.2 ± 6.1	36.0 ± 6.9	35.8 ± 5.8	36.3 ± 6.0
8 min	35.8 ± 5.9	35.8 ± 6.3	35.8 ± 6.0	37.3 ± 6.0	36.1 ± 7.2	36.1 ± 6.3	36.2 ± 6.1
10 min	35.7 ± 5.9	36.0 ± 6.6	36.2 ± 6.0	37.1 ± 6.1	36.5 ± 7.1	35.3 ± 5.8	36.1 ± 6.0
Average	35.8 ± 5.8	35.9 ± 6.1	35.9 ± 5.4	37.1 ± 5.9	35.9 ± 6.7	35.5 ± 5.6	36.0 ± 5.7

Legend: WBV = whole-body vibration; SHS = standing in a static half squat position; SHS + 30% BW = SHS with an additional load of 30% of the body weight; WBV 25/2 = WBV at f = 25 Hz, A = 2 mm; WBV 25/2 + 30% BW = WBV 25/2 with an additional load of 30% of the body weight; WBV 50/4 = WBV at f = 50 Hz, A = 4 mm; WBV 50/4 + 30% BW = WBV 50/4 with an additional load of 30% of the body weight. ^*^ statistically significant difference in CMJ height between 2- and 4-min recovery intervals (*p* = 0.03).

## Results

The sphericity assumption in the Mauchly’s test for CMJ data was fulfilled. Two-way repeated-measures ANOVA revealed a non- significant main effect of interventions on CMJ performance [F(5, 10) = 1.44, η^2^ = 0.42, *p* = 0.29] (Table 1). There was a significant effect of rest intervals on CMJ performance [F(4, 11) = 3.51, η^2^ = 0.56, *p* = 0.044] (Table 1). Post hoc tests showed that a rest interval of 4 min displayed significantly higher CMJ values than a rest interval of 2 min [mean difference = 0.60 cm (CI95% 0.04–1.13 cm), *p*=0.031], while there were no other significant differences across the other rest intervals. However, the calculated SWC (SD × 0.2) was 5.5 × 0.2 = 1.10 cm, which is higher than observed CMJ height mean difference. There were no significant interactions among the interventions and rest intervals.

## Discussion

The purpose of this study was to determine the effects of loaded and unloaded WBV interventions on CMJ height among highly trained karate practitioners. The results indicated non-significant CMJ differences among the WBV interventions employed. However, there was a significant main effect of the rest interval on CMJ performance. Specifically, a rest interval of 4 min resulted in significantly higher CMJ values than a rest interval of 2 min.

In the current study, there were no significant differences in the CMJ observed across intervention schemes. The effect of WBV in athletes may be influenced by individual background, such as the performance level (Washif et al., 2019). Even though participants in our study were individuals with extensive training experience in karate, accustomed to explosive lower body movements such as jumping activities (Ravier et al., 2009), they had a lower CMJ height compared to other international karate athletes. For instance, Loturco et al. (2014) reported CMJ height of 43.2 ± 5.3 cm in a sample of nineteen Brazilian professional karate practitioners, which is approximately 5 cm higher than in the current cohort. However, it is possible that athletes in the current study achieved an optimal state of motor neuron excitability and reflex sensitivity, leading to a reduction in vibration sensitivity (Bullock et al., 2008; Hortobágyi et al., 2015). It is known that lower-level athletes are more responsive and have a greater degree of improvement in jumping performance when exposed to WBV, compared to elite-level and well-trained athletes with comparable training experience (Washif et al., 2019). Consistently, the efficacy of WBV for increasing jumping performance has been demonstrated in recreationally trained men (Turner et al., 2011) and ‘competitive’ and lower-level athletes (Dallas et al., 2015; Washif, 2019). Therefore, additional research is needed to explore alternative WBV schemes that can be beneficial in elite karate athletes.

The external loading in WBV carried out in this study was not influential to generating enhancement in jump performance. For example, a WBV 50/4 at 30% BW (i.e., high frequency vibration and external loads) protocol resulted in the lowest CMJ values compared to other groups. This contradicted previous studies that noted improvement in performance with external loads under WBV (Naclerio et al., 2014; Pojskic et al., 2015; Rønnestad, 2009). Researchers suggest employing WBV with 30 Hz with an amplitude of 4 to 10 mm to elicit benefits in WBV (Dabbs et al., 2012; Washif et al., 2019). A noticeable outcome utilizing a low vibration frequency and amplitude (i.e., 25 Hz, 2 mm) WBV 25/2 + 30% BW protocol was identified in this study, which somewhat coincides with the previous recommendation. It seems that PAPE occurred at different times, highlighting the need for individuality when using loaded WBV. Therefore, it is necessary for coaches and athletes to determine the optimal loading stimulus on a case-by-case basis for designing WBV-based warm-up strategies.

On the other hand, the current study demonstrated an improvement in performance with 4-min recovery duration compared with that of 2-min duration. These data imply that, regardless of vibration stimuli, loaded or unloaded conditions, adequate recovery duration (i.e., 4 min) after the stimulus is more important in maximizing vertical jump performance. For example, Armstrong et al. (2010) discovered a substantial improvement in CMJ performance at the 5^th^ min and the 10^th^ min after different WBV stimuli among untrained participants. Similarly, Adams et al. (2009) observed that a 1- to 5-min interval after WBV was optimum for improving performance. However, McBride et al. (2010) and Dallas et al. (2023) showed that the effect of WBV might last up to 8 min following WBV, which partly supports the current study findings. Interestingly, several authors (Avelar et al., 2014; Washif, 2019) found PAPE effects immediately after the intervention, which might be explained by other factors, such as the influence of athleticism (Washif, 2019). In this study, it seems that a 4-min rest was sufficient to allow recovery from WBV, thereby increasing jump performance. However, it must be acknowledged that the mean difference in CMJ height between the rest intervals of 2 and 4 minutes was lower (i.e., 0.60 cm) than the obtained SWC (i.e., 1.10 cm) and as such it might be questionable how significant it is in practical settings. Nevertheless, the study’s findings suggest a distinct efficacy range for using WBV to enhance CMJ height. However, further research is needed to identify the exact mechanisms contributing to the enhancement in leg power resulting from a rest interval after WBV exposure.

This study has several limitations. First, the study was conducted under acute conditions, which reduces its applicability in long-term settings. Second, interpretation of the results may be limited to the participants or comparable cohort athletes. Additionally, the warm-up protocols employed did not include baseline measurements, which are crucial to elucidate information regarding PAPE. Moreover, the performance of multiple jumps with 2-min rest intervals might influence the CMJ outcomes of this study. Exploring multiple jumps at longer rest intervals (e.g., 5 min) may potentially reduce residual effects. Future studies addressing these limitations are warranted.

In conclusion, utilizing a 4-min rest interval irrespective of the preconditioning schemes may have potential for enhancing jumping performance among highly trained karate practitioners. Both the loaded and unloaded WBV and non-WBV schemes used in the current study appear to produce similar effects, regardless of the vibration (amplitude, frequency, etc.) and load variables. As a final note, to optimize the effectiveness of warm-ups and introduce training variety, it is advisable for coaches and athletes to consider implementing the vibration stimulus employed with a 4-min recovery period.
